# Supplementary material for: Dietary diversity and its correlates among pregnant adolescent girls in Ghana
Source: PLoS One. 2021 Mar 8;16(3):e0247979. doi: 10.1371/journal.pone.0247979 (PMC7939348; doi:10.1371/journal.pone.0247979)
Supplement: Attachment — Submitted filename: REVIEWER 2 RESPONSE.docx [file pone.0247979.s001.docx]

Department of Biochemistry and Biotechnology,

College of Science, KNUST,

Kumasi, Ghana.

The Editors-in-Chief,

PLOS ONE.

**POINT-BY-POINT REVISIONS IN RESPONSE TO REVIEWER COMMENTS**

Dear Editors-in-Chief,

Thanks for reviewing our manuscript.

In line with the reviewer’s comments, the following revisions have been made to the Manuscript ID: PONE-D-20-16523 entitled **“Dietary diversity and its correlates among pregnant adolescent girls in Ghana”** by Linda Afriyie Gyimah, Reginald Adjetey Annan, Charles Apprey, Odeafo Asamoah-Boakye, Linda Nana Esi Aduku, Wisdom Azanu, Herman E. Lutterodt, and Anthony Kwaku Edusei.

Response to Reviewer Two Comments

Abstract

Point 1: Although a 3-day repeated 24-hour dietary recall data were collected, the results reported in the manuscript are based on only the first recalls according to your methods. Therefore, it is misleading to report that the analyses presented in the manuscript were based on a 3-day 24-hour recalls.

**Response 1**: This point has been rectified. It now reads: “As part of a larger longitudinal cohort of 416 pregnant adolescents, the FAO and FHI Minimum Dietary Diversity for Women was used to determine dietary diversity score of the participants from a previous days’ 24-hour dietary recall data.” Please refer to lines 18 to 21.

Point 2. Report the mean age of the girls.

**Response 2**: The mean age of the girls has been reported. Please see line 24

Point 3. The odds ratios were estimated in a multivariate logistic regression model so should be reported as adjusted.

**Response 3:** Both Unadjusted and Adjusted odds ratio have been used in the revised manuscript. Please see Table 6.

Introduction
Point 4. You report that dietary diversity and its correlates in pregnant adolescent girls have not been studied in Ghana, but what have studies conducted elsewhere on pregnant adolescent girls found?

**Response 4:** Thank you for the suggestion. Information on studies on dietary diversity of pregnant adolescents have been inserted into the manuscript. Please see lines 96-105.

Point 5. Paragraph 4, line 6: Correct grammar in the sentence: “Pica, another form of craving involves …”.

**Response 5:** The sentence has been corrected. It now reads; “Pica practice is a form of craving which involves the ingestion of non-food substances like clay, soap, ashes, paint, paper among others”. Please see line 85-86.

Point 6. Paragraph 4, line 8: Correct grammar in the sentence: “It is useful in protecting against pathogens …”

**Response 6:** The sentence has been corrected. It now reads “It is useful for defence against pathogens and toxins, quelling nausea, vomiting, and diarrhoea.” Please refer to lines 87-88

Point 7. Your study is premised on the fact that improving dietary diversity will contribute to desirable pregnancy outcomes so it will be good to provide some statistics to back this.

**Response 7**: Thank you for the suggestion. This has been duly inserted. Please see lines 99-109

Point 8. The last sentence “This study assessed …” should include “Region” after “Ashanti”.

**Response 8**: The point has been inserted. Please see line 125.

Methods
Study setting
Point 9. First paragraph, line 4: Revise the sentence “Each district has a …” to “Each district has a district hospital, health centres and several Community-based Health Planning Services (CHPS) compounds.”

**Response 9:** Revision has been inputted. Please see lines 119-110

Study design
Point 10. First paragraph, line 2: The sentence “The study involves 416 pregnant adolescents with gestational age up to 32 gestational weeks …” should be revised to “The study involves 416 adolescents with gestational age up to 32 weeks …”

**Response 10**: Thank you for the correction. This point has been revised using the average gestational week of antenatal clinic attendance of the participants. Please see line 136.
Data collection
Point 11. Line 1: The sentence “A standardized questionnaire were …” should be revised to “A standardized questionnaire was … ”

**Response 11**: Thank you for the correction. The sentence has been corrected. Please see line 186.

Point 12. Line 3: The sentence “Data on age and parity …” should end with “books” and not “book”.

**Response 12**: The sentence has been corrected. Please refer to line 188.

Point 13. The last sentence is not complete.
Assessment of dietary diversity and eating behaviour

**Response 13:** This section has been rewritten. Please see lines 196-203.

Point 14. Line 3: The sentence “The FAO’s Women’s Dietary Diversity (WDDS) …” should be “The FAO’s Women’s Dietary Diversity Score (WDDS) …”

**Response 14:** Thank you for the correction. We are now using the recent Minimum Dietary Diversity for Women (MDD-W) instead of the Women’s Dietary Diversity Score (WDDS). Please refer to lines 227-240.

Point 15. Line 7: Correct grammar in the sentence: “The first 24-hour recall …”

**Response 15**: This point has been rewritten to improve its clarity. Please see line 234.

Point 16. It is not clear how the WDDS was calculated, please explain the scoring used.

**Response 16:** This comment has been rectified in point 14. How the MDD-W was calculated has been inserted. Please see lines 222 – 240.

Point 17. The adequate category (5-9) of WDDS should include a score of 10.
Assessment of food availability and poverty

**Response 17**: This comment has been rectified in point 14. The new categories have been inserted.

Point 18. Line 3: Correct grammar in the sentence “Scores between <2 means …”

**Response 18**: Thank you for the correction. The sentence has been corrected. It now reads; “Scores <2 means little or no hunger, 2 – 3 means moderate hunger, and 4 – 6 means severe hunger.” Please refer to lines 217-218.

Point 19. Last sentence: Check the categories of lived poverty index scores.

**Response 19**: The lived poverty index categories have been revised. Please see line 224

Data analysis
Point 20. The sentence “Data were first entered into Microsoft Excel 2019 and thoroughly cleaned to eliminate errors in the data.” should be revised as “Data were first entered into Microsoft Excel 2019 and thoroughly cleaned to eliminate errors.”

**Response 20**: Thank you for the correction. It has been inserted. Please see line 247-248.

Point 21. Line 3: Start the sentence with a capital letter (S).

**Response 21**: The correction has been inserted. Please see line 249

Point 22. Line 5: The sentence “Descriptive statistics was …” should be revised to “Descriptive statistics were …”

**Response 22**: The correction has been duly implemented. Please see line 251-252

Point 23. Line 10: The sentence “A non-parametric …” should be “Non-parametric tests … were ….”

**Response 23:** The correction has been duly implemented. Please see line 258

Results
Point 24. Paragraph 1, line 3: I do not think the sentence “More rural than urban participants were younger teenagers (8.1% versus 7.4%, p=0.853) …” is accurate. This is because although 8.1% is more than 7.4%, the difference in the rates can be explained by chance given the insignificant p-value. The same applies to paragraph, line 4: Sentence “More participants who were practising …”

**Response 24**: This point has been corrected. It now reads; “Though not significant, a greater proportion of rural than urban participants were younger teenagers (8.1% versus 7.4%, p = 0.853) and were employed (31.2% versus 26.3%, p = 0.321).” Please see lines 271-273. “More participants who were practising …” this sentence has been revised, please see lines 290-291

Point 25. Table 1: The category of income “Between 500” should be “Between 100 and 500”.

**Response 25:** The correction has been duly implemented. Please see Table 1

Point 26. Table 3: Please specify in the data analysis section why Fisher’s exact test was used.

**Response 26**: Fisher’s exact test was used to report p-values of cross tabs analysis in which either the cells in the variables were less than 5 or the variables were 2×2 variables. For example, Community Type has two categories (Urban and Rural) and was compared to Dietary Diversity which also has 2 categories.

Point 27. Table 3: While I agree with the use of Chi-square test for education and Fisher’s exact test for income, it is not clear to me why Fisher’s exact test was used for community type, age group, marital status, occupation, and parity.

**Response 27**: The table has been revised. Chi-square test was used for variables with more than two variables (Income and Education) whilst Fisher’s exact test was used for variables with two categories (Age, Community type, Parity, Marital status and Occupation).

Point 28. Table 4: The variable “Low Poverty Index” should be corrected to “Livid Poverty Index”.

**Response 28**: Thank you for the correction. The correction has been inserted accordingly. Please refer to table 4.

Point 29. The heading “P VALUE” should be “X2, P VALUE”.

**Response 29**: Thank you for the correction. The correction has been inserted accordingly. Please refer to table 4.

Point 30. Table 4: It is not clear why Fisher’s exact test was used for food aversion, food craving and pica practice.

**Response 30**: Fisher’s exact test is used for variables with two categories. Since Food aversion, Pica and Food Craving were categorised into 2, Fisher’s exact test was used

Point 31. Table 6: Please use adjusted odds ratio instead of odds ratio.

**Response 31**: Thank you for the suggestion. This table has been revised accordingly. Please see table 6.

Point 32. Table 6: Marital status is missing from this table.

**Response 32**: This table has been revised. Marital status has been inserted into the table. Please see table 6.

Point 33. Table 6: The 95% confidence interval, 1.1-136.5, for the category of income “More than 500” is too wide. You can consider merging the categories “Between 100-500” and “More than 500” to rectify this.

**Response 33**: Thank you for the suggestion. This table has been revised. Please see table 6.

Point 34. Why did you calculate Relative Risks for bivariate analyses (Tables 3 and 4) and Odds Ratios for multivariate analysis (Table 6) for the same set of variables? For comparison, I will prefer this same measure of association at both levels.

**Response 34:** Thank you for the correction. This table has been duly revised. Please refer to table 6.

Discussion
Point 35. First sentence: I think you studied dietary factors (food aversion, food craving and pica practice) in addition to socio-demographic factors.

**Response 35**: The suggestion has been duly implemented. Please see lines 376-377

Point 36. Paragraph 2, line 2: In the sentence “More than half …” I do not think the DDS was for the last 3 days.

**Response 36**: Thank you for the correction. This sentence has been revised. Please see line 379

Point 37. Paragraph 2, line 6: “A study in …” should be “Studies in …”

**Response 37:** The suggestion has been effected. Please see line 383

Point 38. Paragraph 2, line 9: Revise the sentence “Our study, the others in Ghana, and others compared outside Ghana, …”

**Response 38**: The sentence has been corrected. Please see lines 386

Point 39. Paragraph 3, line 4: Revise the sentence “A possible explanation is that improve …”

**Response 38:** The sentence has been revised. Please see line 438.

Yours Sincerely,

Linda Afriyie Gyimah

(Corresponding Author)
